# Vaccination of patients with hematological malignancies: barriers and strategies to improve uptake

**DOI:** 10.3389/fmed.2026.1911976

**Published:** 2026-09-28

**Authors:** Toni Valković, Paolo Salamon, Vanja Vasiljev, Tomislav Rukavina

**Affiliations:** 1 Specialty Hospital Medico Rijeka, Rijeka, Croatia; 2 Department for Medical Oncology and Hematology, Division for Internal Medicine, General Hospital Pula, Pula, Croatia; 3 Faculty of Health Studies, Sveuciliste u Rijeci, Rijeka, Croatia; 4 Department of Social Medicine and Epidemiology, Sveuciliste u Rijeci Medicinski Fakultet, Rijeka, Croatia; 5 Teaching Institute of Public Health of Primorje-Gorski Kotar County, Rijeka, Croatia

**Keywords:** barriers, hematology, immunity, immunocompromised, vaccination

## Abstract

Vaccination is one of the most successful developments in the history of medicine, having saved countless human lives. Although vaccination is of the utmost importance for patients with hematological neoplasms, it can also be challenging due to the often reduced or absent serological responses. Despite vaccination, breakthrough infections may occur among patients with hematological malignancies, particularly in those with impaired vaccine-induced immune responses. Although humoral responses are frequently impaired, vaccine-induced cellular immunity is preserved in a substantial proportion of patients with hematological malignancies and may contribute to protection against severe infections. Maximizing the immune response to vaccination requires optimal timing of vaccination relative to the administration of antineoplastic therapy. Patients may also fear vaccine side effects and worry that vaccination might influence their cancer therapy. Finally, some medical professionals, family members, and caregivers are insufficiently aware of the importance of vaccination for patients with hematological diseases, as well as their own vaccination. Despite significant developments in the field of vaccination of immunocompromised patients, addressing these challenges will require further high-quality clinical and translational research; continuous education of patients and healthcare providers; and enhanced cooperation and coordination among primary care practitioners, and epidemiological and hematological departments.

## 1 Introduction

Patients with malignant hematological diseases are immunocompromised (1) for several reasons (Table 1). Firstly, hematological tumors affect immunocompetent cells—such as T and B lymphocytes (including plasma cells), neutrophils, macrophages, and antigen-presenting cells—thereby weakening their physiological defense functions. Secondly, the therapies used to treat hematological neoplasms—including standard chemotherapy, targeted drugs, and newer immunotherapy modalities lead to short- or long-term damage and depletion of different immune system components. Thirdly, patients with hematological tumors are often elderly individuals whose immune systems may already be compromised by age and frequent accompanying comorbidities.

**Table 1:** Causes of increased risk of infection in hematological patients.

Causes of increased risk of infection in hematological patients	
Underlying Disease	Direct suppressive effects on innate and acquired immunity (immunocompetent cells, hypogammaglobulinemia)
Applied Therapy	Direct suppressive effects on innate and acquired immunity (immunocompetent cells, hypogammaglobulinemia) Damage to natural barriers (skin, mucous membranes)
Patient Age	Immunity naturally compromised by the aging process
Comorbidities	Accompanying diseases further compromise the immunological functions and resistance to pathogens

In patients with hematological neoplasms, infections are the second most common cause of death, following only death from the malignant disease itself. Infections also frequently lead to treatment delays, which negatively impact treatment outcomes and the possibility of achieving remission, and increase the number and length of hospitalizations and the overall treatment costs (2). For example, invasive pneumococcal disease occurs with a 60-fold higher incidence in patients with hematological cancer compared to the healthy population (3). Some studies show that the risk of death from invasive pneumococcal disease is highest among patients with multiple myeloma or chronic lymphocytic leukemia (4). Furthermore, the influenza risk is six-fold higher in patients with multiple myeloma compared to the general population (5). The influenza-attributable mortality rate can reach 9% among hospitalized hematological patients (6), and up to 33% in immunocompromised patients undergoing chemotherapy, compared with 3% among controls (7).

The COVID-19 pandemic has brutally highlighted the severity of secondary immunodeficiency in persons with hematological diseases. Patients with hematological tumors exhibited a mortality rate exceeding 30%, which was higher than that among patients with solid malignancies, and almost double the rate in the general population with COVID-19 (8–10). The largest epidemiologic clinical study, EPICOVIDEHA, reported a 31% mortality rate among over 3,000 patients with active hematological malignancy during the first waves of the pandemic (11).

Many innovative drugs, particularly various immunotherapy modalities, are being increasingly used in earlier lines of treatment for hematological diseases; which will likely lead to further decreases of mortality. Some currently incurable hematological diseases may even become curable, at least for some patients. These improvements are likely to have unintended consequences, including deeper and longer-lasting immunosuppression and an increased number of various infections.

Vaccination is among the greatest achievements in the history of medicine, having saved countless human lives. For patients with hematological tumors, vaccination (e.g., against SARS-CoV-2, influenza, pneumococcus, meningococcus, *Haemophilus*, diphtheria, tetanus, pertussis, human papilloma virus, hepatitis A and B, chickenpox, herpes zoster, measles, rubella, mumps, respiratory syncytial virus) must be a mandatory part of supportive care, and performed in a timely manner according to the international and national guidelines or the recommendations of health institutions.

This manuscript was prepared as a narrative review of the literature on vaccination in adult patients with hematological malignancies. A literature search was performed in PubMed/MEDLINE for studies published from 1989 through 2025 (most of the included publications date from the last five years). Articles published in English were eligible for inclusion. The literature search focused on studies evaluating vaccine immunogenicity, effectiveness, safety and vaccination strategies in adults with hematological malignancies. Priority was given to prospective studies, randomized clinical trials, meta-analyses, and contemporary clinical guidelines. When available, the most recent evidence was preferentially cited. Animal studies, preclinical studies, pediatric-only studies, conference abstracts without full-text publication, duplicate publications, and articles not directly relevant to the review topic were excluded. Studies were selected based on their relevance to the objectives of the review, methodological quality, and clinical significance, with priority given to recent evidence.

Unlike previous reviews addressing vaccination in broader populations of immunocompromised patients with cancer, this review focuses exclusively on patients with hematological neoplasms. Although recent systematic reviews and meta-analyses have assessed vaccine immunogenicity and clinical outcomes in this population, our aim is to provide an integrated, clinically oriented overview of the main challenges related to vaccination and potential strategies to overcome them, rather than to discuss the technical indications and schedules of individual vaccines. The relatively greater emphasis on COVID-19 vaccination in this review reflects the current evidence base, as most recent high-quality studies investigating vaccine efficacy, immunogenicity, and safety in patients with hematological malignancies have focused on SARS-CoV-2 vaccines. However, an effort was made to include other vaccines used in hematological patients.

## 2 Humoral response rates after vaccination in different hematological neoplasms

The patients with hematological tumors often show lower vaccine-induced antibody responses compared with the general population, due to damage and reduced numbers or function of immunocompetent cells that contribute to the generation of an immune response after vaccination. Importantly, seroconversion and antibody titers are measures of vaccine immunogenicity and should not be automatically interpreted as evidence of clinical protection unless a validated correlation of protection for the specific vaccine and pathogen has been established.

In a study of patients with hematological malignancies, Monkman et al. reported a 21% seroconversion rate after H1N1 influenza vaccination, which was significantly higher than that in unvaccinated patients (0%), but significantly lower than that previously reported in healthy adults (12). In their systematic review of the effects of influenza vaccination in patients with hematological diseases, Le Torre et al. analyzed pooled data from 22 studies, including 19 cohort studies, 2 randomized clinical trials, and 1 cross-sectional study from 1993 to 2016. For H1N1, they reported a 30% protection rate after a booster dose. For H3N2 and influenza B, the pooled protection rates after the first vaccination dose were 42.6% and 39.6%, respectively. The response rates following a booster dose were 35% for H1N1, 23% for H3N2, and 29% for influenza B (13).

Several studies have demonstrated that patients with multiple myeloma exhibit an impaired ability to achieve a good humoral response following vaccination. Notably, vaccination against pneumococcus has yielded a satisfactory response in 33%–60% of patients, and similar responses have been reported after vaccination against influenza and COVID 19 (14–16).

Additionally, following vaccination against pneumococcal polysaccharide, patients with chronic lymphocytic leukemia exhibit an antibody response rate ranging around 50%–60%, which is lower compared to controls (17). Among patient with chronic lymphocytic leukemia, studies have also shown that better immunogenic results are achieved with conjugate vaccines compared to polysaccharide vaccines, since conjugate formulations are more immunogenic. Patients who exhibit an adequate antibody response tend to have less-advanced disease stages, higher gamma-globulin levels, and lower concentrations of soluble CD23 (17–19). Additionally, pneumococcal conjugate vaccines reportedly induce an antigenically broader, and more durable, polyfunctional antibody response, even in patients undergoing hematopoietic stem cell transplantation (20, 21).

Frederiksen et al. have demonstrated that antibody responses were comparable between patients with Hodgkin's disease before therapy and the healthy population but were significantly attenuated in patients with Hodgkin's disease years after completion of therapy (22). In splenectomized patients with non-Hodgkin lymphoma, Petrasch et al. found that vaccination with pneumococcal polysaccharides elicits an adequate antibody response in 45.4% of cases. However, among patients who do not respond, revaccination does not lead to any further increase in pneumococcal antibody levels (23).

Einarsdottir and colleagues performed a single-center retrospective study of vaccination in hematological patients treated with CAR T-cell therapy (24). Among patients without seroprotection before vaccination, the response rates were highest for tetanus and polio (100%), moderate for diphtheria (75%) and *Haemophilus influenzae* type b (62%), and lower for pertussis (48%), hepatitis A (43%), hepatitis B (44%), and pneumococcal disease (33%). Recipients of CD19 CAR T-cells showed higher rates of pre-vaccination seroprotection compared to BCMA recipients; however, the post-vaccination responses did not significantly differ between these groups (24).

Following hematopoietic stem cell transplantation (HSCT), vaccine-induced immune responses vary considerably according to the type of vaccine and the degree of immune reconstitution. The highest vaccine responses have been reported for *Haemophilus influenzae* type b (71%–100%) and pneumococcal conjugate vaccines (68%–99%), whereas the polysaccharide vaccine alone is less immunogenic but broadens serotype coverage when administered after completion of the conjugate vaccine; 20%–84% for influenza vaccines; 40%–94% for hepatitis B vaccines; and approximately 65%–85% for meningococcal vaccines. Recombinant herpes zoster vaccine (RZV) induces robust cellular and humoral immunity and has demonstrated significant clinical protection against herpes zoster despite only moderate antibody responses. In contrast, evidence regarding Human Papillomavirus (HPV) vaccination remains limited (25).

A prospective study conducted by the Croatian Cooperative Group for Hematological Disease (KROHEM) demonstrated that humoral responses to mRNA COVID-19 vaccination significantly improved after the second dose, although response rates varied according to the underlying hematological disease. The poorest seroconversion rates were observed in patients with lymphoma and chronic lymphocytic leukemia, whereas patients with myeloproliferative neoplasms, particularly chronic myeloid leukemia, achieved the highest seroconversion rates (26).

A few systematic reviews and meta-analyses have established key findings across large patient populations (27–29). Compared to the general population, patients with hematological diseases exhibit significantly lower seroconversion rates after vaccination against COVID-19, with reported values of 30–33.3% after the first vaccine dose, and 62–65.3% after the second dose (27, 28). Piechotta et al. reported optimal immune responses ranging from 38.1–99.1% following the initial two vaccine doses, based on the first assessment performed at least 14 days after administration of the second dose (29).

However, all these findings should be interpreted with caution, as substantial heterogeneity in study populations, underlying diseases and treatments, vaccine platforms, immunological assays, timing of assessment, and subgroup composition limits direct comparisons across studies and introduces uncertainty regarding the generalizability of reported response rates.

Overall, the results of listed investigations indicate substantial heterogeneity in vaccine-induced antibody responses according to the underlying hematological disease and treatment. Response rates were highest in patients with myeloproliferative diseases and lowest among those with chronic lymphocytic leukemia, as well as in patients receiving anti-CD20 monoclonal antibodies or BTK inhibitors. Furthermore, patients who underwent allogeneic or autologous HSCT have generally exhibited higher response rates than those treated with CAR T-cell therapy.

## 3 Cellular response rates after vaccination in different hematological neoplasms

Although humoral responses after vaccination are frequently impaired in patients with hematological malignancies, cellular immunity appears to be relatively preserved in a substantial proportion of patients.

Studies evaluating COVID-19 vaccines have demonstrated detectable T-cell responses in a substantial proportion of patients with hematological malignancies, including those with lymphoid malignancies and recipients of HSCT or CD19-directed CAR-T therapy. Reported rates of cellular responses range from approximately 33% to 86% among patients with hematological malignancies and from 42% to 72% among HSCT/CAR-T recipients (30). Importantly, several studies have shown that T-cell responses may develop despite impaired antibody production. In patients with B-cell depletion, including those receiving anti-CD20 therapies, more than 70% were able to mount detectable cellular immune responses following SARS-CoV-2 vaccination, suggesting that cellular immunity may provide an additional component of vaccine-induced immunity despite inadequate humoral responses (31). Haggenburg et al. demonstrated that most patients with hematological malignancies, including individuals with chronic lymphocytic leukemia, lymphomas, and recipients of CD19-directed CAR-T therapy, developed robust cellular responses after a two-dose mRNA vaccination regimen against SARS-CoV-2, further supporting the relative preservation of cellular immunity in this population (32). Consistent with these findings, treatment with anti-CD20 antibodies, BTK inhibitors, or BCL-2 inhibitors appears to have a substantially greater impact on antibody responses than on T-cell immunity (33). Interestingly, patients lacking detectable humoral responses have demonstrated increased frequencies of spike-specific CD8+ T cells compared with individuals with preserved antibody responses, suggesting a potential compensatory immune mechanism, although the extent to which this translates into clinical protection remains uncertain (34). Importantly, studies in which humoral and cellular responses were assessed in parallel provide evidence that these two components of vaccine-induced immunity may be partially dissociated in cancer patients. In a prospective study of patients with hematological malignancies who received mRNA-1273, humoral and IFN-*γ*-mediated cellular responses were detected after the second dose, but a significant discrepancy was observed among patients who received anti-CD20 therapy, in whom only 17.5% developed a humoral response compared with 71.1% who demonstrated a cellular response (35). A prospective study of cancer patients receiving active treatment assessed antibody and IFN-*γ* responses after the first and second doses of vaccine and directly compared BNT162b2 with the inactivated whole-virion vaccine BBIBP-CorV. Both vaccines induced cellular and humoral responses, but BNT162b2 produced stronger cellular responses, and a positive correlation was observed between IgG antibody titers and T-cell responses regardless of vaccine platform (36). These findings support the concept that cellular immunity can be preserved despite an impaired humoral response, but they also indicate that the relationship between antibody and T-cell responses is not uniform. It is important to note that the available evidence should therefore not be interpreted as evidence that cellular immunity fully compensates for deficient antibody responses. Instead, humoral and cellular immunity should be considered complementary components of vaccine-induced protection, and their relative contributions likely depend on the underlying disease, treatment, vaccine platform, and time of assessment.

The clinical relevance of cellular immunity is supported by observations that higher circulating CD8+ T-cell counts are associated with improved survival among patients with hematological malignancies and COVID-19, including those with B-cell depletion (37).

However, cellular responses are influenced by the underlying disease and treatment status. Patients with multiple myeloma may exhibit impaired CD4+ T-cell responses, potentially related to corticosteroid exposure and anti-CD38-directed therapies (38). Nevertheless, booster vaccination has been shown to enhance cytokine-producing T-cell responses in this population, and additional vaccine doses can further improve SARS-CoV-2-specific cellular immunity in patients with hematological malignancies (39).

Differences in vaccine platforms have also been observed. While mRNA vaccines generally induce stronger antibody responses than vector-based vaccines, both platforms are capable of generating cellular immunity (32). In patients with hematological malignancies, some studies have suggested stronger T-cell responses following mRNA-1273 vaccination compared with BNT162b2 vaccination (40). In the general population, comparative analyses of different vaccine platforms have demonstrated that all evaluated vaccines can induce durable memory CD4+ T-cell responses. These findings suggest that cellular immune memory may persist beyond the period during which circulating antibody levels decline, although the duration and clinical significance of such cellular immunity in patients with hematological malignancies remain incompletely defined (32).

Although most available data on vaccine-induced cellular immunity originate from SARS-CoV-2 studies, T-cell responses have also been demonstrated following vaccination against other pathogens. Among these, the RZV has provided the strongest evidence for robust cell-mediated immunity, including in immunocompromised individuals and patients with hematological malignancies, where cellular responses appear to be better preserved than humoral immunity. Influenza vaccines also induce antigen-specific CD4 + and CD8+ T-cell responses, although clinical data regarding their protective role remain less extensive, while evidence for clinically relevant cellular immunity after hepatitis B and pneumococcal vaccination is more limited (40, 41).

Evidence on cellular immunity remains limited, particularly in non-Western cohorts, with few longitudinal studies evaluating humoral and T-cell responses across vaccine doses or comparing different vaccine platforms. Further prospective studies integrating detailed immunological assessments with clinical outcomes are needed to clarify the contribution of cellular immunity to protection against severe infection and to optimize vaccination strategies in this vulnerable population.

## 4 Breakthrough infection in vaccinated patients with hematological neoplasms

The above-described results indicate that active infections occur after vaccination in many patients who are undergoing treatment for malignant hematological disease. However, there is a lack of data regarding breakthrough infections (with diseases other than COVID-19) in patients with hematological malignancies.

Pagano et al. investigated 1,548 cases of breakthrough COVID-19 infections in adult patients with hematological malignancies, who had received at least one dose of an anti-SARS-CoV-2 vaccine between January 2021 and March 2022. They concluded that although mortality was significantly reduced compared to in the pre-vaccination era, breakthrough COVID-19 in this population was still associated with considerable mortality (42). Similarly, in a retrospective cohort analysis of almost 6,000 hematological patients, Mittelman et al. showed that vaccinated individuals with hematological neoplasms (particularly those undergoing active treatment) experience worse outcomes from breakthrough COVID-19 infections, compared to vaccinated individuals with an intact immune system (43).

Wang et al. reported a 13.4% cumulative risk of breakthrough infection among patients with hematological malignancies, ranging from 11.0% in acute lymphocytic leukemia to 17.2% in multiple myeloma, and 17.4% in chronic myeloid leukemia, compared to a significantly lower 4.5% risk among controls. Moreover, hematological patients who experienced breakthrough infections exhibited a 37.8% overall risk of hospitalization, which was markedly higher than the 2.2% risk observed in those without breakthrough infections. Accordingly, the overall mortality risk was 5.7% in patients with breakthrough infections, compared to 0.8% in those without. Together, these findings indicate that, even among fully vaccinated individuals, the risk of breakthrough infections is significantly higher in patients with hematological tumors compared to those without cancer, and that such infections are associated with substantial clinical consequences, including increased rates of hospitalization and mortality (44).

A prospective, multicenter, registry-based cohort study conducted in Spain included 1,394 patients with hematological disorders and reported that 37 (2.6%) developed breakthrough SARS-CoV-2 infection at a median of 77 days (range 7–195) after full vaccination. Most patients were asymptomatic (19/37, 51.4%), while 19% developed pneumonia, and the overall mortality rate was 8%. In multivariate logistic regression analysis, the absence of detectable antibodies at 3–6 weeks after complete vaccination emerged as the only factor associated with the occurrence of breakthrough infection (45).

Piñana et al. also reported that 266 (17%) of 1,551 hematological patients developed breakthrough COVID-19 infection, occurring at a median of 86 days (range 7–391) after full vaccination. Multivariate analysis showed higher rates of breakthrough infection in patients with chronic lymphocytic leukemia (29%) and in those receiving corticosteroids (24.5%), and lower incidence rates in female patients (15.5%) and in those whose last therapy was more than one year ago (14%). Median antibody titers at multiple time-points were significantly lower in patients with breakthrough infections, compared to those without. The cohort exhibited a low SARS-CoV-2-related mortality rate of 1.9% (46).

## 5 Response to vaccination in patients with hematological neoplasms, according to antineoplastic drugs

In their review, Fattizzo et al. have comprehensively summarized all recent data regarding the impact of different drugs on responses to COVID-19 vaccinations, and other vaccines in the era of targeted therapies (47). Many of the novel targeted therapies used for hematologic disease treatment can exert on-target or off-target immunomodulatory effects, which may influence responses to COVID-19 vaccines, and other vaccines. Agents that predominantly affect B cells—such as anti-CD20 monoclonal antibodies, BTK inhibitors, and anti-CD19 CAR T cells—have the most pronounced negative impacts on seroconversion after vaccination, including with vaccines against COVID-19, influenza, pneumococcus, tetanus, and diphtheria toxoid (47). In contrast, JAK2 inhibitors, BCL-2 inhibitors, and hypomethylating agents may reduce immune function, but they appear to have less marked effects on the humoral response to vaccination (47). On the other hand, vaccine efficacy does not seem to be significantly compromised by tyrosine kinase inhibitors used to treat chronic myeloid leukemia and some anti-myeloma therapies, such as proteasome inhibitors and immunomodulatory agents (47). However, reduced seroconversion rates have been observed in patients treated with anti-CD38 and anti-BCMA monoclonal antibodies. Complement inhibitors used for complement-mediated hematologic disorders, and immunosuppressive therapies used for aplastic anemia, generally do not alter seroconversion rates; however, the overall magnitude of the immune response may be diminished in patients receiving steroids or anti-thymocyte globulin (47).

## 6 Effectiveness of booster doses in patients with hematological neoplasms

Recent studies of vaccination against COVID-19 have documented how booster doses impact the overall immune response after vaccination in immunocompromised patients with hematological tumors (Table 2). Huang et al. demonstrated that among 68 patients with hematologic neoplasms (including lymphoma, chronic lymphocytic leukemia, multiple myeloma, and acute and chronic myeloid leukemia), 89.7% achieved seroconversion following booster vaccination. Multivariate analysis revealed that effective neutralization was independently predicted by the absence of B-cell-targeted therapy within six months before vaccination, and higher immunoglobulin levels. The authors concluded that most patients with hematologic malignancies can achieve seroconversion after booster doses; however, ongoing B-cell depletion and hypogammaglobulinemia were negative predictive factors associated with poorer neutralizing responses (48).

**Table 2:** Effectiveness of COVID-19 booster doses in patients with hematological malignancies.

Effectiveness of COVID-19 booster doses in patients with hematological malignancies		
Study	Patient population	Seroconversion
Huang et al. (48)	Various hematological malignancies	89.7%
Shapiro et al. (49)	Various hematological malignancies	56%
Šušol et al. (50)	Various hematological malignancies	23%
Lim & Ahearne (51)	B-cell NHL (No anti-CD20 in 12 months)	92%
	B-cell NHL (anti-CD20 in 12 months)	17%

**Table 3:** The main reasons for COVID-19 vaccine hesitancy among cancer patients (77).

Reasons for COVID-19 vaccine hesitancy among cancer patients
Doubts about the effectiveness of the vaccine
Fear that the vaccine may interfere with and/or delay cancer treatment
Vaccine safety concerns
Beliefs regarding inadequate development of the vaccine
Perception of low subjective risk regarding the disease

Shapiro and colleagues reported that administration of one booster vaccine dose increased the response rate to 56% in patients with hematological malignancies. Prior anti-CD20/BTK inhibitor therapy was associated with reduced vaccine efficacy (49). In another study, Šušol et al. reported that 23% (14/61) of patients with initial failure achieved seroconversion after the third dose, with the worst result in the group of patients with chronic lymphocytic leukemia (50). Finally, Lim and Ahearne showed that among patients with B-cell non-Hodgkin lymphoma, the administration of a third vaccine dose led to improved humoral responses in 92% of those who had not received anti-CD20 therapy within the previous 12 months, compared to only 17% of those who had (51). Patients with indolent B-cell non-Hodgkin lymphoma also exhibited enhanced humoral responses after the third dose; however, approximately one-third still exhibited relatively low antibody levels. It was concluded that although a third (booster) dose can partially improve immune responses, a substantial proportion of patients with NHL continue to show lower antibody levels compared with healthy individuals (51).

Collectively, these findings indicate that booster vaccination can substantially enhance vaccine-induced humoral responses in a proportion of patients with hematological malignancies. The benefit is particularly limited in patients receiving B-cell-depleting or other B-cell-targeted therapies.

## 7 Benefits of vaccination for patients with malignant hematological neoplasms

The numerous studies showing weaker immune responses to vaccination in hematological patients, even after administration of a booster dose (especially in some diseases and after certain therapies), raise questions regarding whether some patients with hematological malignancies benefit from vaccination at all. In a Cochrane systematic review, Cheuk et al. assessed the efficacy of viral vaccines in immunocompromised patients with hematological malignancies, including five randomized controlled trials evaluating inactivated influenza vaccines. This systematic review concluded that, despite attenuated serological responses, influenza vaccination was associated with reduced infection rates and fewer hospitalizations, supporting its routine administration in this high-risk population (52).

Rossi et al. examined factors associated with mortality among hematological patients over 65 years old during the COVID-19 pandemic and found that both vaccination status and the number of vaccine doses received had significant impacts on 90-day survival (53). The 90-day survival probability progressively increased in patients who received zero, one, two, three, or four doses, with statistically significant differences observed across all pairwise group comparisons (53). Thus, the benefit of vaccination should not be assessed solely by antibody responses, but also by clinically meaningful outcomes, including prevention of infection where demonstrable, reduction in disease severity, hospitalization, and mortality among vaccinated compared with unvaccinated patients. Accordingly, the large EPICOVIDEHA survey and other international studies have demonstrated that prioritizing large-scale SARS-CoV-2 vaccination contributed to a reduction of COVID-19-related mortality, decreasing it from approximately 30% in the pre-vaccination era to around 10%–15% after vaccination was introduced (54–57). This shift may be partly related to the more favorable and less aggressive biological behavior of the virus itself, but it is certainly also partly due to patient vaccination and increasing numbers of vaccine doses (57, 58). However, as the presented data shows, a substantial proportion of patients with hematological neoplasms develop measurable vaccine-induced antibody responses following vaccination and, when indicated, booster doses. The likelihood and magnitude of such responses vary substantially according to the underlying malignancy, previous and ongoing anticancer therapy, timing of vaccination and other patient-related and disease-related factors. Nevertheless, the available clinical evidence supports vaccination as an important component of infection prevention in this high-risk population.

## 8 Optimal vaccination timing in patients with hematological neoplasms

Whenever feasible, patients should receive up-to-date seasonal vaccines and other recommended vaccinations 2–4 weeks before treatment initiation, to allow for an adequate immune response. Prior to vaccination, it is necessary to check the patient's previous vaccination status, and data regarding previous VZV infection/VZV serology and HBV serum markers. Notably, if vaccination cannot be administered within this optimal timeframe due to the urgency or ongoing nature of cancer therapy, it should still be strongly recommended early during treatment (59). Therefore, it is highly important that as soon as a hematological malignancy is suspected, the hematologist immediately refers the patient to receive vaccination, in parallel with performing diagnostic procedures and staging the disease. Unfortunately, in clinical practice, the important vaccination step is often forgotten amid the desire to quickly complete the diagnostic processes. A systematic review and meta-analysis suggested that morning vaccination may enhance antibody responses in adults aged ≥60 years, although these findings were derived from immunocompetent populations. Whether optimizing vaccination according to circadian rhythms improves vaccine immunogenicity or clinical protection in patients with hematological malignancies remains uncertain (60).

## 9 Vaccination of medical staff and household contacts of patients with hematological neoplasms

Healthcare workers in direct contact with patients or their materials should be vaccinated according to national guidelines or institutional policies, with different vaccines against a range of pathogens, including MMR, BCG, hepatitis B, VZV, pertussis, influenza, COVID-19, and tetanus/diphtheria/polio. Likewise, household contacts of patients should be vaccinated in accordance with international or national guidelines, as this is an important means of preventing some infectious diseases in hematological patients. For example, the guidelines of the Croatian Cooperative Group for Hematological Diseases (KROHEM) recommends that household contacts receive vaccination against SARS-CoV-2, influenza, measles, rubella, mumps, and chickenpox.

Evidence from cohorts of healthcare workers also provides useful supporting evidence for the relationship between booster vaccination, humoral and cellular immunity, and breakthrough infection. In a prospective study of healthcare workers, a third dose of mRNA vaccine significantly increased both antibody concentrations and the proportion of participants with detectable IFN-*γ* responses; however, cellular responses were not independently associated with protection against subsequent SARS-CoV-2 infection (61). Similarly, a large prospective longitudinal study of healthcare workers who received BNT162b2 after two doses of ChAdOx1 found 100% antibody positivity and 92.8% cellular response positivity one month after booster vaccination, while correlations between antibody responses and IFN-*γ* were weak or negligible, further illustrating that humoral and cellular immunity represent related but partially independent components of vaccine-induced immunity (62). In a separate prospective study of fully vaccinated healthcare workers, breakthrough infections were associated with lower pre-infection neutralizing antibody titers, although most breakthrough infections were mild or asymptomatic (63). Although these studies were conducted predominantly in immunocompetent healthcare workers and therefore cannot be directly extrapolated to hematological patients, they provide relevant evidence that booster vaccination enhances both components of adaptive immunity and that breakthrough infection cannot be predicted based on cellular immunity alone.

Notably, oral polio vaccines and live-attenuated influenza vaccines should not be administered to household contacts or healthcare workers who are in contact with hematological patients suffering from malignant hematological diseases, particularly in cases where patients have recently undergone hematopoietic stem cell transplantation or are suffering graft-versus-host disease (59).

In some countries, vaccination of healthcare workers is not legally mandated but rests on each individual's decision, while in other countries, vaccination of healthcare workers against selected vaccine-preventable diseases is mandated by law or required as a condition of employment, reflecting its recognized role in protecting both healthcare personnel and vulnerable patients. For healthcare workers, the decision to vaccinate should be understood as a professional obligation and always be subordinated to the well-being of the patients. However, there remains a need for healthcare workers to be further educated about the purpose and safety of vaccination. Notably, there were unfounded concerns among healthcare workers that the COVID-19 vaccine could have negative effects on fertility, which highlights the urgent need for education and provision of relevant medical facts and truthful information. The importance of vaccinating healthcare workers should be extensively discussed, as an ethical and practical issue, during the education of doctors and nurses at their faculties and schools.

## 10 Barriers to vaccination of patients with hematological neoplasms related to knowledge, attitudes, and beliefs

Among patients with hematological tumors, the rates of vaccination against various bacterial and viral agents are still not as high as we would like, despite the fact that vaccines are generally available and reimbursed in Western countries. There are many reasons for this state of affairs, including issues related to knowledge, attitudes, and beliefs among both patients and healthcare workers.

One of the most important steps leading to increased vaccination of hematological patients is providing patients with better information about the benefits of vaccination. These patients must receive relevant information from their hematologist immediately after the diagnosis has been made, or after HSCT or CAR-T cell therapy has been performed. It is crucial to take sufficient time to clearly explain to the patient all of the risks associated with infections (morbidity, mortality, delay of therapy, and impact on quality of life) and theimportance of vaccination for preventing possible infections, i.e., what specific benefit the patient receives from vaccination. During this conversation, vaccination should be advocated as the paramount supportive measure that will lead to a successful outcome of the treatment of a malignant disease. To this end, it is important that the doctor presents as much concrete information as possible describing the benefits of vaccination, rather than making general claims. It is also extremely important to explain the safety profiles of individual vaccines. Research conducted among southern rural veterans with cancer in the USA revealed that vaccine hesitancy was largely due to questions about safety, inadequate information, and not seeing the purpose of the vaccine (64). Patients should not be left poorly informed about the importance of vaccination due to a lack of time. The patient should be provided with adequate information to be convinced to undergo vaccination based on their own decision, and not on the authority of the doctor, as this can lead to an anti-vaccine position in some patients (65). Individuals with cancer represent a distinct patient group and may worry that vaccination could adversely influence the effectiveness of their treatment, making this an important issue to address in discussions with these patients. An examination of attitudes towards COVID-19 vaccination conducted among cancer patients in Australia revealed that over one-third of participants were concerned that vaccination might influence their cancer therapy (66). The same study reported that major factors encouraging vaccination were the perceived risk of COVID-19 infection (for themselves and others), and interaction with a trusted healthcare professional. In contrast, notable obstacles included worries related to vaccine development, safety, and potential side effects. Overall, most concerns about vaccination appeared to arise from misinformation, rather than a general opposition to vaccines (66, 67). In addition to speaking with individual patients, it is very important to educate the target population in larger groups. Research findings show the usefulness of continuously educating both patients and healthcare workers about the importance and benefits of vaccination, which is critical for the overall success of a vaccination program in the community (68). Good frameworks for such outreach can include patient associations or meetings of healthcare professionals at hematology departments. A recent global review examined adult attitudes toward influenza vaccination and found that distributing educational materials significantly impacts the acceptance of vaccination by healthcare system users (66). Additionally, within the healthcare system (in this case, within the hematology community), several tools have been proven useful for increasing the number of vaccinated patients—for example, the preventive vaccination checklists, built-in electronic reminders and decision-making tools, and educational initiatives (69).

The existence of international vaccination guidelines for patients with hematological neoplasms is of great importance. National hematology societies may adopt or adapt existing international recommendations to promote timely and standardized vaccination practices. A comparison of the scope and key features of current international vaccination guidelines for patients with hematological malignancies is presented in Table 4 (59, 70–72). Although current guidelines are broadly concordant, important differences exist in the timing and strength of recommendations. Vaccination is generally preferred before the initiation of immunosuppressive therapy whenever feasible, whereas anti-CD20 therapy may substantially impair humoral vaccine responses, leading most guidelines to recommend vaccination before treatment or postponement until immune reconstitution when clinically appropriate. Following HSCT, systematic revaccination is recommended because previous vaccine-induced immunity is generally not considered durable. In CAR-T recipients, vaccination strategies remain less standardized, with timing individualized according to cellular therapy, B-cell recovery, and ongoing immunosuppression. For pneumococcal vaccination, current recommendations increasingly favor conjugate vaccines. International guidelines are broadly concordant in recommending two doses of recombinant zoster vaccine (RZV) for adults with cancer but differ mainly in age thresholds and the recommended timing of vaccination in relation to immunosuppressive therapy, particularly anti-CD20 treatment, HSCT, and CAR-T therapy, while the strength of evidence remains more limited in highly immunosuppressed populations. RSV vaccination is a newer recommendation for selected older and/or high-risk patients, but evidence specifically in hematological malignancies remains limited. Live-attenuated vaccines are generally contraindicated during significant immunosuppression and should only be considered after careful assessment of immune recovery (59, 70–72). Alternatively national societies may develop and regularly update country-specific recommendations that reflect local epidemiology, healthcare infrastructure, vaccine availability, and regulatory policies while remaining aligned with current evidence. All relevant experts, especially epidemiologists and family physicians, should be involved in the process of establishing guidelines, as they are part of the team involved in the vaccination of patients.

**Table 4:** Comparison of the main focus and distinguishing features of current international vaccination guidelines for patients with hematological malignancies.

Comparison of the main focus and distinguishing features of current international vaccination guidelines for patients with hematological malignancies	
Guideline	Main focus/distinguishing features
ASCO	General vaccination recommendations for adults with cancer; emphasis on vaccination before treatment whenever possible; include a guideline for RSV vaccination.
NCCN	Practical recommendations integrated into supportive oncology care, including patients receiving targeted therapies and immunotherapy; include a guideline for RSV vaccination
ECIL/EBMT	Most detailed recommendations for HSCT recipients and patients receiving cellular therapies, including revaccination schedules
CDC/ACIP	National adult immunization schedule, including recommendations for immunocompromised adults and newly licensed vaccines (e.g., a detailed guideline for RSV vaccination)
IMWG	Disease-specific recommendations addressing vaccination in patients with multiple myeloma, particularly those receiving modern immunotherapies (e.g., bispecific antibodies)

To our knowledge, there is presently no mature data regarding whether cancer patients are vaccinated to the same extent as the rest of the population, or whether hematologists themselves have doubts about vaccination. During the COVID-19 pandemic, the Croatian Cooperative Group for Hematological Diseases made substantial efforts to increase vaccination uptake among hematological patients, who generally expressed acceptance of vaccination. Similarly, 79.1% of cancer patients in an Australian study reported that their physicians had recommended vaccination (73). However, COVID-19 vaccine hesitancy among cancer patients varied widely across studies, from 82.1% in Hong Kong to 2% in Ireland, with a mean of 38.4% (74–76). Butow et al. identified several reasons for vaccine hesitancy, with approximately one in five patients being hesitant and many expressing concerns about potential interactions between vaccination and their cancer (77). Greater vaccine acceptance was associated with no cancer recurrence, a longer time since diagnosis, and better quality of life (78), while better performance status was also associated with greater willingness to vaccinate (79). Higher health literacy and education levels were consistently associated with lower vaccine hesitancy (74, 80, 81).

The above text attempted to outline the main challenges related to vaccination of patients with hematological neoplasms, as well as possible ways to overcome them which are summarized in Table 5. Finally, limited research has examined the vaccination of hematological patients from the perspective of the patients or healthcare professionals. There remains a need for new studies in the future to provide us with new evidence and guide us towards the activities and changes in behavior that will best promote vaccination in this vulnerable population.

**Table 5:** Main challenges in the vaccination of patients with hematological neoplasms and possible solutions to overcome them.

Main challenges in the vaccination of patients with hematological neoplasms	Possible solutions to overcome them
Lower seroconversion rates and lower rates of achieving protective antibody titers	Vaccinate at the optimal point in the treatment cycle, preferably before immunosuppressive therapy when feasible Development of next-generation vaccine platforms with improved immunogenicity and more effective humoral and cellular responses after primovaccination	
Weaker immune response following booster dose administration	Individualize booster timing according to the patient's disease, treatment, and degree of immunosuppression Development of next-generation vaccine platforms with improved immunogenicity and more effective humoral and cellular responses after booster doses	
Higher incidence and poorer outcomes of breakthrough infections	Maintain vaccination but combine it with a layered prevention strategy, such as vaccination of household members, caregivers, and healthcare workers, and providing clear instructions regarding early approach to infection diagnosis and treatment	
Frequent untimely vaccination of patients	Integrating vaccination into mandatory procedures (algorithms) during disease diagnosis and staging before starting therapy or after certain forms of therapy Integration of digital health technologies, including electronic vaccination registries, electronic medical record alerts, automated vaccine reminder systems, etc.	
Insufficient knowledge and implementation of international guidelines Lack of transparent national or institutional guidelines	Following and implementation of international guidelines in clinical practice or adoption of national/institutional guidelines based on international ones, but taking into account some local specificities regular updating of adopted guidelines	
Insufficient vaccination rates among healthcare workers, family members, and caregivers	Provide convenient access to vaccination within hospitals and hematology clinics Continuous education and demonstration of the benefits of vaccination in these groups	
Inadequate collaboration among hematologists, epidemiologists, and family physicians	Establish a multidisciplinary vaccination pathway with clearly defined responsibilities	
Numerous barriers related to the knowledge, attitudes, and beliefs of patients and healthcare professionals	Provide patients and healthcare professionals with education/training on the benefits and safety of vaccines, taking into account misinformation	
Insufficient patient education (inadequate number of public health campaigns)	Use multiple channels: brochures, hospital websites, social media, patient organizations, and public-health campaigns	
Insufficient pre- and post-graduate education for physicians and nurses regarding the vaccination of patients with hematological neoplasms	Provide continuing medical education and regular hospital-based training	

## 11 Recent advances and future directions in vaccination of patients with hematological neoplasms

Vaccine development is rapidly evolving, with several novel approaches aiming to overcome the limitations of conventional vaccine platforms, particularly in the immunocompromised population. High-dose, adjuvanted, and recombinant vaccines have emerged as promising strategies to enhance vaccine-induced immune responses in patients with hematological malignancies. The strongest clinical evidence currently supports established non-live vaccines, particularly RZV, for which clinical efficacy has been demonstrated in patients with hematological malignancies. In a phase 3 randomized trial including adults with hematological malignancies receiving or having completed anticancer therapy, RZV induced robust humoral and cellular immune responses and was well tolerated, with a *post-hoc* estimate of vaccine efficacy against herpes zoster of 87.2% (82). These findings provide direct clinical evidence supporting RZV as an effective preventive strategy in this population. Similarly, pneumococcal, influenza, and COVID-19 vaccines have substantial immunogenicity and, for some vaccines and clinical settings, clinical effectiveness data supporting their use, although vaccine-induced immune responses and the magnitude of clinical benefit may vary according to the underlying disease and treatment. Respiratory syncytial virus (RSV) has recently emerged as another important vaccine-preventable pathogen in patients with hematological malignancies, in whom RSV infection is associated with substantial morbidity and mortality (83). In contrast with RZV vaccine, evidence for RSV vaccination in patients with hematological malignancies remains limited. Pivotal RSV vaccine trials largely excluded severely immunocompromised patients, and the ASCO systematic review identified no eligible studies specifically evaluating RSV vaccination in adults with cancer (59). Current recommendations for RSV vaccination in immunocompromised adults therefore rely predominantly on age- and risk-based recommendations and extrapolation from the general population and other high-risk groups rather than on direct efficacy data in hematological malignancies. Emerging studies in transplant populations provide encouraging immunogenicity data, but further evidence is required to define the optimal timing, vaccine formulation, and clinical effectiveness in hematological malignancies (84).

The development of next-generation vaccine platforms has further expanded opportunities to improve immune protection in immunocompromised individuals. Advances in antigen engineering, novel adjuvants, and delivery systems, including nanoparticle-based technologies, aim to enhance both humoral and cellular immune responses. Multivalent and mosaic vaccine designs may broaden immune coverage by targeting multiple pathogen variants or conserved antigenic regions. Although these approaches remain largely under clinical investigation, they represent promising strategies for improving vaccine effectiveness in patients with impaired immunity.

mRNA technology represents one of the most important recent advances in vaccinology. The successful implementation of mRNA vaccines against SARS-CoV-2 has demonstrated the advantages of this platform, including rapid development, scalable manufacturing, and adaptability to emerging pathogens. Compared with conventional vaccine technologies, mRNA vaccines allow rapid modification of antigen sequences and support the development of multivalent formulations, making them particularly attractive for pathogens characterized by antigenic variability. In addition, their ability to induce both antibody-mediated and cellular immune responses provides a strong rationale for further investigation in patients with hematological malignancies, in whom immune responses to traditional vaccines are frequently compromised (85). Beyond currently available vaccines, research is expanding toward novel preventive strategies against respiratory pathogens for which licensed vaccines are not yet available, including human metapneumovirus and parainfluenza virus. Several candidate vaccines (including combined vaccines) based on mRNA, protein subunits, and virus-like particle platforms are currently under clinical evaluation (86, 87). In addition, mucosal vaccine approaches designed to induce local immune responses at the site of viral entry, including secretory IgA and tissue-resident memory T cells, represent a promising strategy for improving protection against respiratory infections, particularly in immunocompromised populations (88, 89).

Future applications of mRNA technology may extend beyond infectious diseases. Personalized neoantigen-based mRNA vaccines are currently being investigated as a novel approach in cancer immunotherapy. By encoding patient-specific tumor neoantigens identified through genomic sequencing, these vaccines aim to induce targeted antitumor T-cell responses and may represent a potential complementary strategy alongside existing immunotherapeutic approaches in hematological and solid malignancies (90).

Another important future direction is the development of precision vaccination strategies. Rather than applying uniform vaccination schedules to all patients, individualized approaches may incorporate disease characteristics, type and timing of anticancer therapy, lymphocyte recovery, immunoglobulin concentrations, and vaccine-specific immune responses. Such strategies may help identify patients who would benefit from additional booster doses, revaccination, or alternative preventive interventions (91).

The increasing complexity of modern anticancer therapies has also highlighted the need for evidence-based vaccination recommendations. In recent years, professional organizations, including the American Society of Clinical Oncology (ASCO), International Myeloma Working Group. National Comprehensive Cancer Network (NCCN), and other international expert groups, have developed consensus recommendations addressing vaccination in patients receiving novel immunotherapy and targeted treatments, such as bispecific antibodies or CAR-T therapy (59, 70, 71). These guidelines provide an important framework for optimizing vaccine timing and improving protection while considering treatment-related immune impairment.

Beyond vaccine development itself, implementation strategies will remain essential for improving vaccine uptake. Digital health technologies, including electronic vaccination registries and clinical reminder systems, as well as integrated and multidisciplinary vaccination clinics, may facilitate identification of vaccination gaps and improve adherence to recommended schedules (92–94). Furthermore, multidisciplinary vaccination programs involving hematologists, infectious disease specialists, nurses, pharmacists, and other healthcare professionals may enhance vaccine delivery and address vaccine hesitancy among patients and healthcare providers.

Overall, future vaccination strategies for patients with hematological malignancies are expected to become increasingly personalized, integrating advances in vaccine technology with detailed assessment of individual immune status. Continued collaboration among hematologists, immunologists, infectious disease specialists, vaccine researchers, and public health organizations will be essential to develop and implement more effective preventive strategies for this highly vulnerable population.

## 12 Conclusion

Patients with hematological malignancies remain highly vulnerable to infections and represent a heterogeneous population with substantial variation in vaccine-induced immune responses. Although vaccination remains a cornerstone of infection prevention, vaccine-induced immune responses are often impaired in patients with hematological neoplasms, particularly in patients with chronic lymphocytic leukemia, and in those receiving B-cell–depleting or B-cell-targeted therapies, including anti-CD20 antibodies, BTK inhibitors, and CAR-T therapy. However, the magnitude of impairment varies according to the several factors, such as underlying disease, disease activity, treatment exposure, timing of vaccination, and degree of immune dysfunction. Among currently available vaccines, RZV currently has the strongest direct evidence for clinical efficacy in patients with hematological malignancies, while pneumococcal, influenza, and COVID-19 vaccines are supported by substantial immunogenicity data and, in several settings, evidence of clinical benefit. Evidence remains limited regarding other vaccines, newer vaccine platforms, and optimal vaccination strategies for patients receiving novel cellular or targeted therapies.

Future high-quality clinical and translational studies should therefore prioritize clinically meaningful outcomes, including infection, hospitalization, severe disease, and mortality, in addition to humoral and cellular immunogenicity, to better define vaccine effectiveness and facilitate the development of individualized and more effective vaccination strategies for this vulnerable population.
